# *STITCHER 2.0*: primer design for overlapping PCR applications

**DOI:** 10.1038/srep45349

**Published:** 2017-03-30

**Authors:** Damien M. O’Halloran, Isabel Uriagereka-Herburger, Katrin Bode

**Affiliations:** 1 Department of Biological Sciences, The George Washington University, Science and Engineering Hall 6000, 800 22nd St. N.W. Washington DC 20052, USA; 2 Institute for Neuroscience, The George Washington University, 636 Ross Hall, 2300 I St. N.W. Washington DC 20052, USA; 3 The School Without Walls, 2130 G St. N.W., Washington, DC 20037 USA

## Abstract

Overlapping polymerase chain reaction (PCR) is a common technique used by researchers in very diverse fields that enables the user to ‘stitch’ individual pieces of DNA together. Previously, we have reported a web based tool called *STITCHER* that provides a platform for researchers to automate the design of primers for overlapping PCR applications. Here we present *STITCHER 2.0*, which represents a substantial update to *STITCHER. STITCHER 2.0* is a newly designed web tool that automates the design of primers for overlapping PCR. Unlike *STITCHER, STITCHER 2.0* considers diverse algorithmic parameters, and returns multiple result files that include a facility for the user to draw their own primers as well as comprehensive visual guides to the user’s input, output, and designed primers. These result files provide greater control and insight during experimental design and troubleshooting. *STITCHER 2.0* is freely available to all users without signup or login requirements and can be accessed at the following webpage: www.ohalloranlab.net/STITCHER2.html.

Overlapping PCR is a common technique used by molecular biologists in various fields that allows the user to ‘stitch’ together various individual DNA fragments^1^. Some of the applications of overlapping PCR include gene synthesis by assembly PCR, reporter gene fusion using GFP or RFP based fluorophores, and detection experiments through techniques such as loop-mediated isothermal amplification (LAMP)^2^,^3^,^4^. We have previously developed a web-based tool called STITCHER that automates the design of primers for many overlapping PCR applications^5^. Here we present a significant upgrade called STITCHER 2.0, which considers more algorithmic parameters during primer design, and provides numerous visual guides and output files that provide a better resolution of the designed primers and starting sequence for the end-user (Fig. 1). Unlike STITCHER, STITCHER 2.0 allows the users to customize more features, and provides more robust control regarding overlap complementarity. STITCHER 2.0 also offers a suite of result features that were not offered with STITCHER; these features include the following: comprehensive tabulated output of primer features that includes complementarity analysis; tabulated output of compatible primer pairings; graphical analysis of the input sequence that highlights the nucleotide composition and also sliding windows of GC% and AT%; horizontal bar chart of designed primer features that includes the average, minimum, and maximum values for primer GC%, *T*_m_, complementarity scores, positions, length, and hairpin values; primer sequence map that highlights the position of each designed primer; and also a facility to draw primers in specific locations along the input sequence, which is particularly useful at targeting exon/intron boundaries. These new features contained within STITCHER 2.0 will design more robust primers, reduce artifacts, and provide better resources during experimental design and troubleshooting.

## Results and Discussion

**Figure 2:**
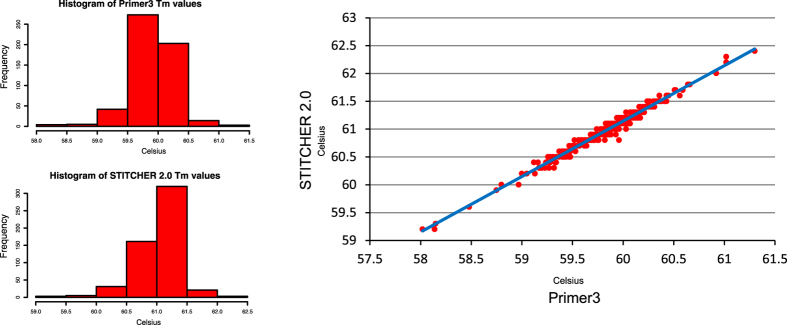
Comparison of primer *T*_m_ values between STITCHER 2.0 and Primer3. Primer *T*_m_ values were compared between STITCHER 2.0 and primer design software, Primer3^10^,^11^. Batch Primer3^18^ was used to generate primer *T*_m_ values for 544 primers and compared with the values calculated by STITCHER 2.0. Correlation of the data sets revealed an R-squared value of 0.98.

**Figure 3:**
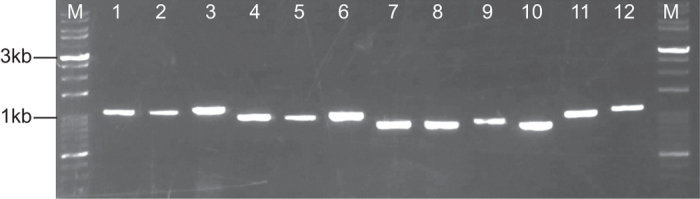
PCR validation experiments using primers designed by *STITCHER 2.0*. Primers were designed using STITCHER 2.0 to span the *ama-1* locus of *Caenorhabditis elegans*. Using genomic DNA as template, robust bands were observed for each primer pair. The marker in the first and last lanes use VersaLadder™ 100-10,000 bp (Gold Biotechnology Inc., St Louis, MO), and the other lanes contained the following primer pairs: 1) 5F 1R; 2) 5F 2R; 3) 5F 3R; 4) 14F 1R; 5) 14F 2R; 6) 14F 3R; 7) 16F 1R; 8) 16F 2R; 9) 16F 3R; 10) 18F 2R; 11) 4F 2R; 12) 4F 3R. The primer sequences were as follows: 4F: CGCAGAATGGAAGAAGAA; 5F: CGCAGAATGGAAGAAGAAC; 14F: TCCAACAGATCACAGACGA; 16F: TGTACCGTACTTCCAGTCCC; 18F: ACTTCCAGTCCCACCACTT; 1R: CCGGTTGGAGGTGAAGAT; 2R: CCCGGTTGGAGGTGAAGAT; 3R: GGTTGACGATTAAAGACGAT.

**Figure 4:**
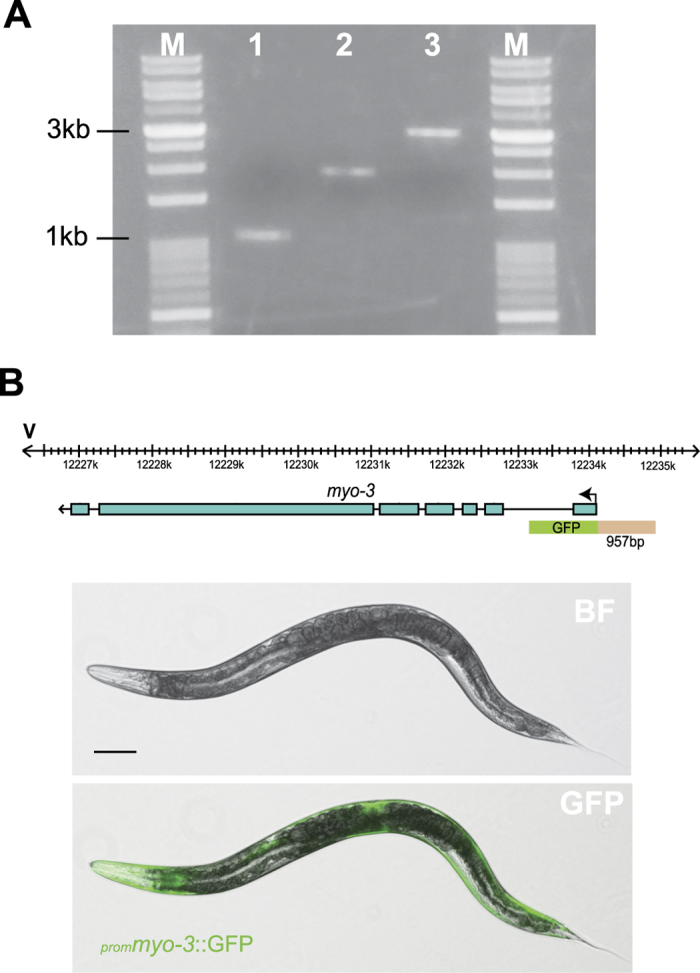
Validation of overlapping primers designed by *STITCHER 2.0*. **(A)** Primers were designed using STITCHER 2.0 to fuse the promoter region of the *C. elegans myo-3* gene to GFP. Using genomic DNA as template, the promoter region of *myo-3* was amplified (2^nd^ lane), and fused to the GFP coding region containing the *unc-54* 3′UTR which was amplified from the plasmid pPD95.75 (3^rd^ lane). To ensure that the correctly fused product was obtained (4^th^ lane), the resulting fusion was PCR purified and directly microinjected in the germline of wildtype (N2) animals and the resulting expression pattern examined from stable transgenic animals. The first and last lanes (labelled M) on the gel use VersaLadder™ 100-10,000 bp marker (Gold Biotechnology Inc., St Louis, MO). **(B)** Cartoon of the gene structure of the *myo-3* gene and promoter region captured for the fusion PCR. The promoter sequence is represented in beige and the GFP coding sequence plus *unc-54* 3′UTR is indicated in green. Stable transgenic lines expressing the ^*prom*^*myo-3*::GFP reporter fusion was imaged, and a representative image of the expression pattern from a day 1 adult is shown (strain DMH109 *Ex*[^*prom*^*myo-3*::GFP]). The expression pattern observed from our promoter GFP fusion matches previous descriptions of the *myo-3* expression pattern in body wall muscle and vulval muscle cells of the hermaphrodite^19^,^20^.

Input for STITCHER 2.0 is a single sequence in fastA format^6^. Sequences can be downloaded from NCBI in fastA or ASN.1 by entering the accession number(s) under the *Get Sequences* section. Once a sequence is inserted, the user completes the *Primer Design Parameters* section. By default, both forward and reverse primers are designed, but either can be deselected. The primer size for the forward and reverse primers can be specified (this size should not include the length of the overlap fragment). The 5′ and 3′ search areas are then specified by the user followed by the maximum and minimum percentage GC and primer *T*_*m*_ values for the forward and reverse primers. Primer *T*_*m*_ values are calculated using parameters defined previously^7^,^8^,^9^ and compare very closely with *T*_*m*_ values calculated by Primer3^10^,^11^ (Fig. 2). Salt concentrations used during primer *T*_*m*_ calculations are set to 50 mM by default but can be changed to 10 mM. The addition of a GC clamp to either the forward or reverse primer can be specified (by default this is set to ‘No’). During primer design STITCHER 2.0 will move along in increments defined by the user within the specified 5′ and 3′ search areas. The default increment is 2 bp, however, the forward and reverse increments can be modified independently to facilitate specific applications. Next, STITCHER 2.0 will perform various calculations to approximate the Gibbs free energy (*ΔG°*) for DNA duplexes and also score the number of complementary matches to return a weighted score for self-complementarity, overhang complementarity, 3′ complementarity, and primer dimer pairs. STITCHER 2.0 sets a cutoff for each of these scores to default values which were determined from our testing (Figs 3 and 4), but can be changed by the user to be more or less stringent. STITCHER 2.0 can also remove N’s (non-designated bases) from the input sequence, and by default will also exclude repetitive sequence from designed primers. Repetitive sequence is defined as a stretch of four or more identical base pairs (e.g. CCCC or TTTT) or dinucleotide repeats (e.g. CGCGCGCG or TGTGTGTG) in a row. Overlapping PCR is often used to generate PCR fusions to fluorophore reporters or sequencing adapters, and so STITCHER 2.0 includes a drop-down selection of pre-defined overlapping fragments for commonly used applications (Table 1). Users can also enter their own overlap oligo in the *user-defined* overlap text box for the reverse overlap. In the case of experiments that use overlapping PCR to introduce mutations to a target sequence in a specific location, the user can enter the forward mutant oligo in the *mutant overlap* text box and STITCHER 2.0 will automatically generate the appropriate reverse oligo, and use the values entered in the 5′ and 3′ search area text boxes to design a single mutant primer pair. Finally, the user can set which output files will be generated by STITCHER 2.0. By default, tables of the forward and reverse primers and a list of compatible primer pairs are returned, but additional outputs can be selected. Users can learn more about each parameter by clicking on the user-guide download link within the *Online Help* tab of the *Features* section. Users can also email queries or suggestions to the address contained within the *Contact* section or enter their email address to receive notifications of updates.

The main advantage of STITCHER 2.0 as compared to other software tools that can facilitate overlapping primer design^12^,^13^,^14^ is that STITCHER 2.0 can be adapted to multiple applications such as PCR fusion or LAMP, offers a very easy to use interface, does not require any additional dependencies apart from a browser, and returns diverse outputs that include tables of primers and their features, graphical analysis of the input sequence and designed primers, interactive primer sequence maps, and the ability for the user to draw primers in specific locations. These features make STITCHER 2.0 novel and provides an intuitive interface to design robust primers, and offers comprehensive resources during experimental design and troubleshooting.

## Methods

The user-interface for STITCHER 2.0 is written using JavaScript, CSS, and HTML5 and deploys the Bootstrap framework (http://getbootstrap.com) and jQuery library (https://jquery.com). Output charts are generated using d3.js (https://d3js.org), Canvas.js (http://canvasjs.com), Fabric.js (http://fabricjs.com), and the neXtProt sequence viewer (https://github.com/calipho-sib/feature-viewer). Server side requests are handled using custom PHP scripts. STITCHER 2.0 was tested successfully on Microsoft Windows 7 Enterprise ver.6.1, Mac OSX El Capitan ver.10.11.5, and Linux Ubuntu 64-bit ver.14.04 LTS, using Safari ver. 10.0, Chrome ver. 53.0.2785.143, Internet Explorer ver. 9.0.8.112.16421, and Firefox ver. 45.0.2.

To validate primers designed using STITCHER 2.0 a series of PCR reactions using primers designed by STITCHER 2.0 that span the *Caenorhabditis elegans* gene *ama-1* were performed using the following cycling conditions: 1) 95 °C for 5 mins; 2) 95 °C for 30 secs; 3) 57 °C for 30 secs; 4) 72 °C for 1 min, with steps 2 to 4 repeated 35 times, followed by 72 °C extension for 10 mins. The resulting products from these PCR reactions are shown in Fig. 3, and in each case bands of the correct size were obtained from each PCR.

To validate overlapping PCR primers designed by STITCHER 2.0, a transcriptional GFP reporter fusion was made by designing primers to fuse the promoter region of the *myo-3* gene in *C. elegans* to GFP as described previously^1^,^15^. The promoter sequence (Fig. 4 – lane 2) was amplified by PCR using Expand High Fidelity polymerase (Roche – product # 04 738 250 001) using the primer pair: myo-3 F: ACTGTTGTTGTGATCTTCTTCGC and fusion primer myo-3: AGTCGACCTGCAGGCATGCAAGCTGTGGTCGTGGGTTTGATG using the following cycling conditions: 1) 94 °C for 5 mins; 2) 94 °C for 30 secs; 3) 58 °C for 30 secs; and 4) 68 °C for 2 mins; steps 2 to 4 were repeated 35 times followed by 68 °C extension for 10 mins. The GFP plus the *unc-54* 3′UTR was PCR amplified (Fig. 4 – lane 3) from the plasmid pPD95.75 (available from AddGene: http://www.addgene.org/1494/) using the primers: GFP-F: AGCTTGCATGCCTGCAGGTCGACT GFP-R: AAGGGCCCGTACGGCCGACTAGTAGG and the following cycling conditions: (1) 95 °C for 5 mins; (2) 94 °C for 30 secs; (3) 55 °C for 1 min; and (4) 72 °C for 2 mins; steps 2 to 4 were repeated 35 times followed by 72 °C extension for 10 mins. Following PCR amplification and purification of the promoter and GFP fragment, a fusion PCR step was performed using 10 ng of each amplicon with the nested primer pair: GFP nested primer: GGAAACAGTTATGTTTGGTATATTGGG and myo-3 F nested: TTGTTGTGATCTTCTTCGCA and the following cycling conditions: (1) 95 °C for 5 mins; (2) 94 °C for 30 secs; (3) 55 °C for 30 secs; and (4) 68 °C for 4 mins; steps 2 to 4 were repeated 35 times followed by 68 °C extension for 10 mins. The resulting PCR product (Fig. 4 – lane 4) was purified and directly micro-injected into the germ-line of wildtype (N2) worms using standard protocols^16^.

For all PCR validation reactions, *C. elegans* gDNA was isolated by collecting worms that spanned all stages in a 1.5 ml tube. Worms were maintained using standard protocols^17^. 200 μl of lysis buffer (60 mg/ml proteinase K, 10 mM Tris-Cl, pH 8.3, 50 mM KCl, 2.5 mM MgCl_2_, 0.45% IGEPAL, 0.45% Tween-20, 0.01% gelatin) was added to the pellet of worms and frozen at −80 °C for 10 mins followed by heating to 60 °C for 1 hr and then heated to 95 °C for 15 mins. Tubes were then centrifuged at 13,000 rpm for 1 min and 50 μl of supernatant isolated for PCR.

## Additional Information

**How to cite this article:** O’Halloran, D. M. *et al. STITCHER 2.0*: primer design for overlapping PCR applications. *Sci. Rep.* **7**, 45349; doi: 10.1038/srep45349 (2017).

**Publisher's note:** Springer Nature remains neutral with regard to jurisdictional claims in published maps and institutional affiliations.

## Figures and Tables

**Figure 1:**
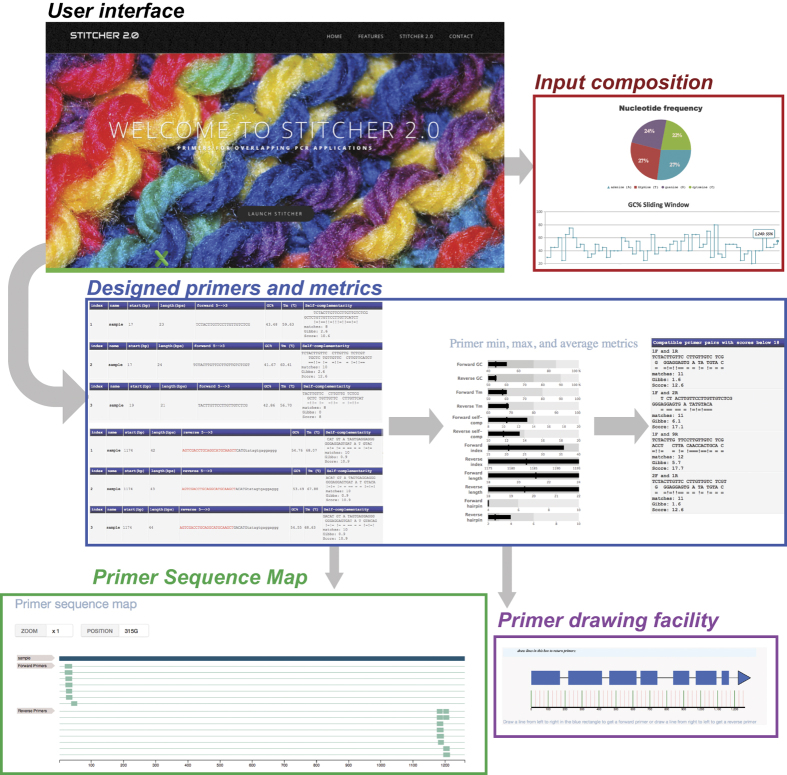
*STITCHER 2.0* interface and output files. The various output files from STITCHER 2.0 are shown. The first file returns a compositional analysis of the input sequence. Next, tables are returned of the designed forward primers, reverse primers, and compatible primers as well as various metrics describing the forward and reverse primers designed by STITCHER 2.0. A graphic of the primer sequence map is also generated, and finally, an intron-exon map of the input sequence is generated which can be used to draw primers in specific locations.

**Table 1:** DNA sequences of predefined overlap fragments.

Primer Name	DNA Sequence 5′ → 3′
GFP	ACAGCTCCTCGCCCTTGCTCACCAT
GFP_Celegans	AGTCGACCTGCAGGCATGCAAGCT
mCherry	TATCTTCTTCACCCTTTGAGACCAT
RFP	TATCTTCTTCACCCTTTGAGACCAT
YFP	ACAGCTCCTCGCCCTTGCTCACCAT
tdTomato	TGACCTCCTCGCCCTTGCTCACCAT
Illumina paired end adapter 1	ACACTCTTTCCCTACACGACGCTCTTCCGATCT
Illumina paired end adapter 2	CTCGGCATTCCTGCTGAACCGCTCTTCCGATCT
Illumina paired PCR primer 1	AATGATACGGCGACCACCGAGATCTACACTCTTTCCCTACACGACGCTCTTCCGATCT
Illumina paired PCR primer 2	CAAGCAGAAGACGGCATACGAGATCGGTCTCGGCATTCCTGCTGAACCGCTCTTCCGATCT
Illumina paired sequencing primer 1	ACACTCTTTCCCTACACGACGCTCTTCCGATCT
Illumina paired sequencing primer 2	CGGTCTCGGCATTCCTACTGAACCGCTCTTCCGATCT
